# Population and Individual Elephant Response to a Catastrophic Fire in Pilanesberg National Park

**DOI:** 10.1371/journal.pone.0003233

**Published:** 2008-09-17

**Authors:** Leigh-Ann Woolley, Joshua J. Millspaugh, Rami J. Woods, Samantha Janse van Rensburg, Robin L. Mackey, Bruce Page, Rob Slotow

**Affiliations:** 1 Amarula Elephant Research Programme, School of Biological and Conservation Sciences, University of KwaZulu-Natal, Durban, South Africa; 2 Department of Fisheries and Wildlife Sciences, University of Missouri, Columbia, Missouri, United States of America; The University of New South Wales, Australia

## Abstract

In predator-free large herbivore populations, where density-dependent feedbacks occur at the limit where forage resources can no longer support the population, environmental catastrophes may play a significant role in population regulation. The potential role of fire as a stochastic mass-mortality event limiting these populations is poorly understood, so too the behavioural and physiological responses of the affected animals to this type of large disturbance event. During September 2005, a wildfire resulted in mortality of 29 (18% population mortality) and injury to 18, African elephants in Pilanesberg National Park, South Africa. We examined movement and herd association patterns of six GPS-collared breeding herds, and evaluated population physiological response through faecal glucocorticoid metabolite (stress) levels. We investigated population size, structure and projected growth rates using a simulation model. After an initial flight response post-fire, severely injured breeding herds reduced daily displacement with increased daily variability, reduced home range size, spent more time in non-tourist areas and associated less with other herds. Uninjured, or less severely injured, breeding herds also shifted into non-tourist areas post-fire, but in contrast, increased displacement rate (both mean and variability), did not adjust home range size and formed larger herds post-fire. Adult cow stress hormone levels increased significantly post-fire, whereas juvenile and adult bull stress levels did not change significantly. Most mortality occurred to the juvenile age class causing a change in post-fire population age structure. Projected population growth rate remained unchanged at 6.5% p.a., and at current fecundity levels, the population would reach its previous level three to four years post-fire. The natural mortality patterns seen in elephant populations during stochastic events, such as droughts, follows that of the classic mortality pattern seen in predator-free large ungulate populations, i.e. mainly involving juveniles. Fire therefore functions in a similar manner to other environmental catastrophes and may be a natural mechanism contributing to population limitation. Welfare concerns of arson fires, burning during “hot-fire” conditions and the conservation implications of fire suppression (i.e. removal of a potential contributing factor to natural population regulation) should be integrated into fire management strategies for conservation areas.

## Introduction

Successful conservation management of large mammals has the ironic consequence of problems associated with overpopulation [1]. This is particularly so with fragmented, small populations or with keystone species that, at high population densities, can impose negative impacts on the system [2]. A key uncertainty that emerges is what limits such populations naturally, and whether such limitation will occur at the same levels in human modified systems (e.g. with fences or artificial water) compared to natural systems [1]. Some species may be resource limited, displaying density dependent responses [3]. Others may be top-down limited by predators [4]. Long-lived species may also be limited by environmental catastrophes, such as drought, flood, fire or disease, which can cause sudden and, at times, significant shifts in population size and dynamics over a very short time, if the effects of such catastrophic impacts on demographics are of sufficient frequency and intensity [5]. Although there is some theoretical and empirical evidence that drought may limit elephant populations [6], there has been no evaluation of the role that fire may play. Due to their rare occurrence, evaluation of the impacts of such events on population dynamics and individual responses is also rare.

Fire is commonly applied for ecosystem management in savannas and arson fires occur regularly [7]. Whilst the impact of fire on plant mortality has been extensively researched, there is little research that has assessed the influence of fire on mortality in animals or the welfare issues associated with fire in savanna systems. Given elephants are highly intelligent and social mammals, fire, or other severe disturbances, may also precipitate behavioural or physiological responses. For example, high elephant poaching caused heavily stressed elephants to form larger groups than unstressed elephants [8].

The extremely hot, dry, windy (“hot-fire”) conditions experienced towards the end of the dry season in Pilanesberg National Park (PNP), a small (570 km^2^), fenced reserve in South Africa facilitated the spread of an uncontrolled wild fire. The area had a 1–2 year fuel load, with the last pre-fire rains falling in May 2005. Below average (∼630 mm p.a.) annual rainfall of 554 mm was recorded during the 2004/5 wet season, while 824 mm fell in 2003/4 and 411 mm in 2002/3. On 21 September 2005, ambient midday air temperature was 34°C, while wind speed was generally strong but variable in direction. The fire entered the western boundary of the Park near Tlatlaganyane village on 20 September 2005 and within two days had moved across an area of approximately 61 km^2^. This catastrophic fire resulted in the mortality of 29 and injury to 18 elephants, unprecedented in PNP, where few natural elephant mortalities had occurred prior to this event [9], [10]. This event provided us with the opportunity to assess the potential influence of severe fires in which animals become trapped on the behavioural, physiological and demographic responses of the elephant population. We provide an assessment of (1) the behavioural and physiological responses of the elephants to this large disturbance event, and (2) the potential for rare, stochastic mass-mortality events to limit population size. We examined movement and herd association patterns of six GPS-collared breeding herds, and evaluated physiological response through faecal glucocorticoid metabolite (stress) levels [11], [12]. We investigated population size, structure and projected growth rates.

## Results

### Behavioural response

#### Daily displacement

**Figure 1:**
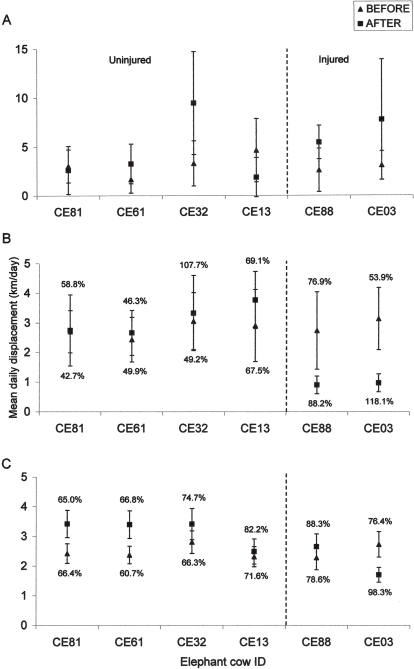
Behavioural response to fire giving mean daily displacement (±95% CL) of each collared cow over: (A) 4 days, (B) 10 days and (C) 3 months, before and after the fire. Coefficients of variation (CV (%)) of daily displacement before or after the fire are given above or below the upper or lower limit of CL bars. Pre-fire CV's are located above if the value of pre-fire mean±95% CL is located above (i.e. is greater than) post-fire mean±95% CL. Where mean±95% CL pre- and post-fire are equal, pre-fire CV's are located below CL bar.

There was no significant difference in mean daily displacement over four days before versus after the fire for all cows (t_5_ = −1.238, *P* = 0.271). However, injured herds (CE03, CE88) and a herd in close proximity to the fire at time of injury (CE32) moved significantly further per day after the fire than before (t_2_ = −6.915, *P* = 0.020), while there was no significant difference in mean daily displacement of uninjured herds (CE13, CE61, CE81) over four days before versus after the fire (Figure 1).

There was no significant difference in daily displacement among collared cows over a ten-day period before the fire (F_5, 54_ = 0.305, *P* = 0.908). However, there was a significant difference in daily displacement among collared cows over the ten-day period, post-flight, after the fire (F_5, 54_ = 9.346, *P*<0.0005). Injured cows (CE03 and CE88) moved at a significantly slower rate in the ten days after the fire (t_9_ = 4.486, *P* = 0.002; t_9_ = 2.756, *P* = 0.022 respectively), compared with ten-day daily displacement before the fire (Figure 1). Uninjured cows CE61, CE81, CE32 and CE13, did not show a significant change in daily displacement in the ten day period before versus after the fire (t_9_ = −0.450, *P* = 0.663; t_9_ = −0.930, *P* = 0.928; t_9_ = 1.084, *P* = 0.307; t_9_ = 0.745, *P* = 0.476 respectively) (Figure 1). There was no significant difference in the coefficients of variation (CV) of daily displacement for the ten-day periods pre- and post-fire (t_9_ = −2.064, *P* = 0.094), but a general trend of increased variability is evident for those herds involved in the fire (CE03, CE88) or those close to the fire when injuries occurred (CE32) (Figure 1).

There was no significant difference in daily displacement among collared cows over a three-month period before the fire (F_5, 540_ = 1.709, *P* = 0.131). However, there was a significant difference in daily displacement among collared cows over a three-month period post-fire (F_5, 540_ = 5.720, *P*<0.001). The daily displacement over three months for injured cows CE03 and CE88, as well as CE88's new matriarch CE13, were not statistically different, while daily displacement for CE88 and CE13 was not statistically different from uninjured cows (CE81, CE61 and CE32) (Figure 1). Uninjured cows CE81 and CE61 showed significant increase in their daily displacement during three months post-fire (t_90_ = −3.664, *P*<0.001; t_90_ = −3.830, *P*<0.001) (Figure 1). Severely injured cow CE03 showed a significant decrease in three-month daily displacement post-fire (t_90_ = −3.240, *P*<0.0005) (Figure 1). Less severely injured cow CE88, matriarch CE13 and uninjured cow CE32 showed no significant difference in three month daily displacement before versus after the fire (t_90_ = −1.337, *P* = 0.185; t_90_ = −0.747, *P* = 0.457; t_90_ = −1.641, *P* = 0.104 respectively) (Figure 1). There was a significant difference between pre- and post-fire CV in daily displacement over three months (t_9_ = −2.984, *P* = 0.031), with a general trend of increase in variability post-fire (Figure 1).

#### Home range

**Figure 2:**
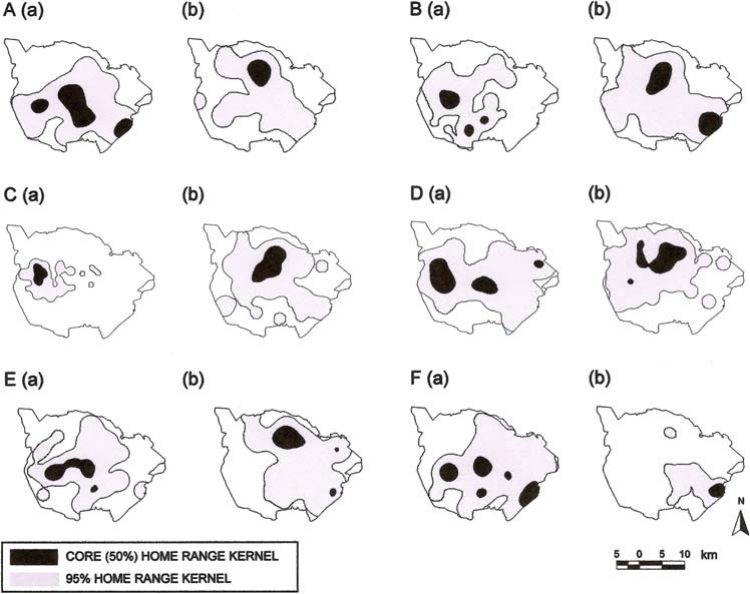
Behavioural response to fire showing core home range and 95% home range kernels for: uninjured adult cows (A) CE13, (B) CE81, (C) CE61, (D) CE32 and injured adult cows (E) CE88 and (F) CE03 three months (a) before and (b) after injury in a fire on 21 September 2005 in Pilanesberg National Park. The percentage overlap between 95% home ranges before and after the fire was: (A) 80.5, (B) 72.7, (C) 49.7, (D) 86.0, (E) 74.1 and (F) 52.5 respectively.

There was no significant difference in home range size before and after the fire among all cows (50% kernel size, t_5_ = 0.505, *P* = 0.635; 95% kernel home range, t_5_ = −0.024, *P* = 0.982). Only severely injured cow CE03 reduced the size of her core home range (36.1 km^2^ to 6.3 km^2^) and 95% home range (305.9 km^2^ to 71.6 km^2^) dramatically after the fire and her home range shifted from the central areas of the Park to the south-eastern wilderness area (Figure 2).

Cows spent significantly more time in the wilderness areas of the Park in the three months after than in the three months before the fire (t_5_ = −4.510, *P* = 0.006). Percentage overlap of home ranges indicated a shift in home range location post-fire (Figure 2).

There was no significant difference in core (50% kernel) range size over the 44 day period before versus after the fire (t_5_ = −1.290, *P* = 0.267). However, the size of 95% kernel home range differed significantly over this time period (t_5_ = −3.753, *P = *0.020), with most cows having a larger 95% home range after the fire. Home range size for the 44 day period after the first spring rains was not significantly different to home range size before the rain (50%: t_5_ = 0.096, *P* = 0.928; 95%: t_5_ = 0.654, *P* = 0.542). Percentage overlap of before rain 95% home range and after rain 95% home range was 47.2%, 80.5%, 73.2%, 90.7%, 64.8% and 78.1% for CE03, CE13, CE32, CE61, CE81 and CE88 respectively. Therefore, all except severely injured cow CE03 had similar 95% home range location before versus after the rain. This suggests that the change in season post-fire was not the reason for the change in 95% home range we observed.

#### Herd fission/fusion

The time spent associating with other herds pre- and post-fire was significantly different for uninjured versus injured cows (t_4_ = −3.675, *P* = 0.021). For the first two months post-fire, fission behaviour was exhibited by injured cows, with CE03 and CE88 spending only 10.3 % and 34.7 % of their time associating with other herds respectively, compared with 91.2 % and 62.2 % respectively before the fire. In the third month post-fire, CE03 exhibited increased fusion behaviour, with association time increasing from 10.3 % to 43.8 % and CE88 joined uninjured collared cow CE13 (permanent association to August 2008). Uninjured cows generally exhibited greater fusion behaviour after the fire.

### Physiological stress response

**Figure 3:**
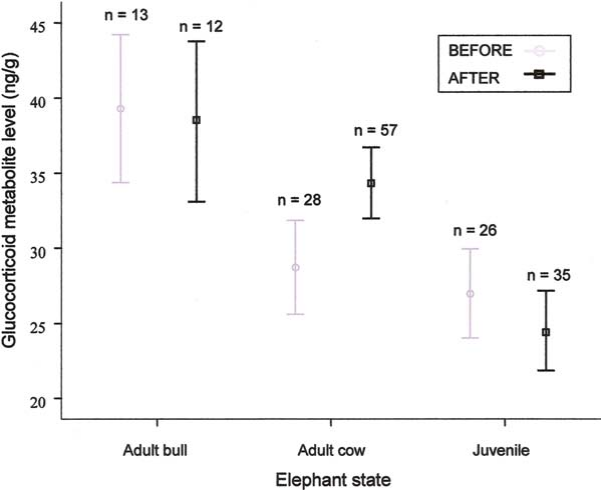
Physiological response to fire indicated by glucocorticoid metabolite (stress) levels (mean±95% CL) for adult bulls, adult cows and juveniles before and after the fire. Sample size (n) is shown above each category.

While pre- versus post-fire measurement, in general, had no significant effect on stress hormone levels (F_1, 133_ = 0.261, *P* = 0.610), there was a significant difference among elephant age-sex classes (i.e. juvenile, adult bull, adult cow) (F_2, 133_ = 16.155, *P*<0.001). There was also a significant interaction between pre- versus post-fire and elephant age-sex class (F_2, 133_ = 4.240, *P* = 0.016). Before the fire, adult cow and juvenile stress levels were not significantly different, but were both significantly lower than adult bull stress levels (Figure 3). Cow stress levels increased significantly post-fire but juvenile and bull stress levels were unchanged by the fire (Figure 3).

Evaluation of stress hormone levels before versus after the first Spring rain fell showed that there was no significant change in stress hormone levels between wet and dry 44 day periods (F_1, 98_ = 0.015, *P* = 0.902).

### Demography

Five family units and six independent adult bulls suffered burn injuries (47 individuals), of which 29 mortalities occurred (17.6% of pre-fire population total) (Table 1). Five juveniles between six and ten years of age, 11 adult females and two adult males recovered from their burn injuries (Table 1). Fifteen infants (≤3 years old), seven weaned calves (4 to 10 years old), four adult females and three adult males died, either as a direct result of their burn injuries, or from euthanasia implemented by Park authorities due to the severity of their injuries (Table 1). Initial post-fire assessment resulted in the euthanasia of two of the four adult females and all three adult males, with veterinarians deciding on strict euthanasia criteria which included >50% burns to total body surface area, marked oedema, eschars, severe supparative oozing and severe impairment of mobility due to burn lesions. Seventeen of the injured juveniles were taken to a holding facility off-site and their wounds treated. Only two of these elephants survived and were released back into the Park. Of the fifteen juveniles that died, ten were euthanazed, with euthanasia criteria including >50% burns to total body surface area, large skin surface area with open tissue, comparative behavioural records indicating severe pain and distress, collapse without recovery after revival, as well as low blood protein and calcium. Euthanasia was only considered in cases where recovery was impossible (criteria for recovery see [13]) and thus mortality can be considered representative of natural fire mortality.

**Table 1:** Demographic response to fire: elephant mortality and survival recorded after a fire in Pilanesberg National Park on 21 September 2005, giving the herd of origin and an estimate of elephant age are given (as of December 2005).

Herd	Collared cow	Age of individuals in different categories *	
		Injured and died	Injured and survived
Gold	CE 57	1, **2**, **3**, 4, 8	**4**, **8**, 8, **10**, **25**, **30**, **35**
Monica	CE 98	1, **2**, 4	**10**, **12**, **30**
Red	CE 07	**2**, 2, 3, **35**	8, **12**
Sheena	CE 88	2, 2, **3**, 4, 4, 6, **30**, **30**	8, **20**
Yellow	CE 03	1, **1**, **2**, 4, 5, **30**	**15**, **42**
Adult bulls		12, 15, 20	12, 15

* Females are indicated in bold.

**Figure 4:**
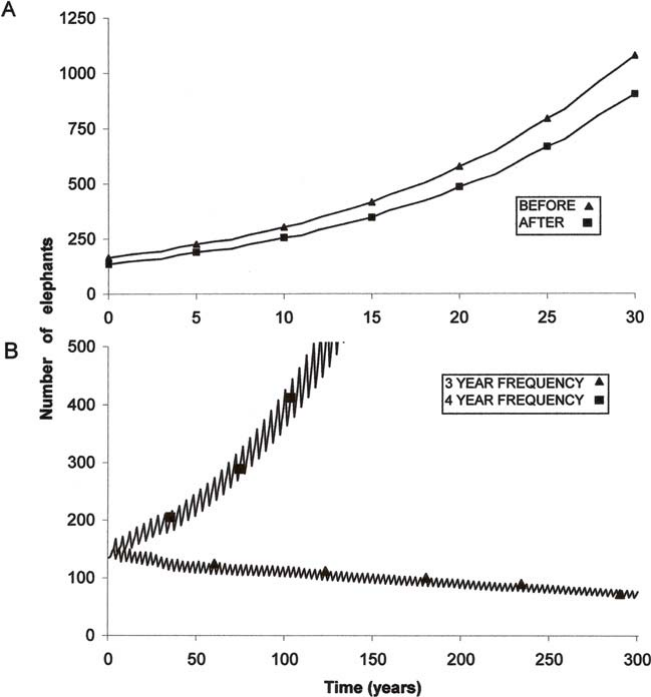
The effect of fire on the future Pilanesberg National Park elephant population: (A) comparative modelled projection over a 30 year period using population data before and then after the fire in September 2005; (B) effect of three year and four year fire frequency on population size over a 300 year period using population data before the fire, and the mortality parameters associated with this fire event.

Age structure, classified according to 10-year and 4-year age classes, was significantly different after the fire than before (10-year age classes: G_3_ = 70.637, *P*<0.001; 4-year age classes: G_3_ = 71.598, *P*<0.001). Model projections over 30 years showed no change in projected population growth rates achieved using demographic data before the fire, as well as demographic data after fire mortalities were accounted for (6.5% p.a.). It took four years for the projected population to recover to the pre-fire population size of 165 individuals (Figure 4). In the absence of fire, the population was projected to grow to 303, 577, and 1079 individuals in 10, 20, and 30 years respectively (Figure 4). Taking into account the effects of fire on the population structure, the population was projected to reach 255, 485, and 903 individuals in the same timeframes (Figure 4). For this type of mortality event to reduce long-term population growth rate to 0%, it would be required at a frequency of every three to four years (Figure 4).

## Discussion

A large disturbance event causing catastrophic injury and mortality has consequences that can significantly affect the functioning and behaviour of an elephant population. In response to a catastrophic fire in PNP, injured elephant cows showed an initial short-term (lasting about four days) flight response post-fire, a longer-term (over about ten days to three months) decrease in daily displacement, a shift in home range, social withdrawal, seclusion to non-tourist areas, and significantly higher stress levels. However, behavioural responses were not limited to injured individuals alone. Uninjured cows also showed altered physiological and behavioural responses post-fire. These cows had significantly raised stress levels, a general increase in daily displacement, more variability in daily distance moved, withdrawal to non-tourist areas and a herd fusion response. Injured herds therefore may have signalled their distress to uninjured herds. Elephant family groups that show a high frequency of association have been known to act in a co-coordinated manner, due to the complex social behaviour and long-range communication used by elephants [14], [15]. The stress of injury, together with social disruption due to the loss of and injury to family members is likely to have affected the behaviour of injured breeding herds. This is additional to the increased vulnerability of injured juveniles to predation, or the compromised ability of injured adult cows to protect their young, which would have increased stress levels. The incidence of elephant calf predation has been found to increase during times of drought when nutritional stress and dehydration facilitates the circumstances where calves can lag behind the herd and become vulnerable to predators [16]. Injured calves were seen alone in PNP after the fire (pers. obs.), increasing their vulnerability to predation.

The physiological and behavioural responses apparent in the PNP population post-fire are consistent with elephant reactions to stressful conditions. Breeding herds showed raised stress levels and a fusion response to cow immobilizations and high-volume tourist activity in PNP [9]. Working elephants in a safari operation had high stress hormone levels associated with transportation and episodic loud noises, such as lightning and thunderstorms and human-induced activities, with baseline levels of faecal glucocorticoid metabolites for adult elephants in PNP of approximately 25 ng.g^−1^ [12]. Heavily stressed elephants, responding to high levels of poaching, formed larger groups than unstressed elephants [8]. Stress responses to culling in Kruger National Park were initial flight, taking elephants outside of home ranges [17], as well as the movement of elephants into and out of culling regions in response to culling events [18], [19]. As these studies indicate, elephants are stressed by human-induced and natural disturbances. Stressed animals alter their behaviour in an attempt to eliminate the stressor. Thus, shifts in home range and seclusion to non-tourist areas are predictable, adaptable responses to disturbance. Therefore, a fire event resulting in elephant mortality has the potential to induce severe behavioural and physiological stress responses (see [20] review of trauma effects on neuroendochrinological development of elephants, and subsequent non-normative behaviour). Whereas drought may cause elephant mortalities over an extended period of time [21], fire mortality occurs within a short time period after the event. Long-term elephant behavioural response to fire mortality may therefore persist, due the dramatic and traumatic nature of the event [20].

The demographic impact of fire on the PNP elephant population predominantly involved the mortality of juveniles (76% of total mortality). Among large herbivore populations where predators are absent, high temporal variation in juvenile survival is often seen, with fairly constant adult survival [22]–[24]. In systems where large predators are present, both adult and juvenile survival responds to environmental variability, due to interactions between resource availability, population size and predation pressure [4]. Without constant predation pressure, the natural mortality patterns often seen in African elephant populations during stochastic events, such as droughts, follows that of the classic mortality pattern seen in predator-free large ungulate populations, which mainly involves juveniles [21], [25], [26]. Fire therefore functions in a similar manner to other environmental catastrophes.

Population structure prior to the fire was significantly different post-mortality, due to predominant mortality in the juvenile age-class. In effect, the loss of a high proportion of juveniles serves not only to lower population size, but also to increase calving interval where the lost calf creates a gap between siblings. However, among large herbivore populations, population growth is most sensitive to adult mortality, especially that of prime-aged females [22]–[24], [29]. The mortality of only four adult females from the PNP population as a result of the fire meant that projected population growth rate remained unchanged. Therefore, the ability of this type of stochastic, catastrophic mortality exhibited in the PNP fire to limit population size or growth would require higher or more frequent mortality, would need to include a higher proportion of adult females [19], [29], or cause demographic delays such as a decline in conception rates, increased inter-calving interval, or increased age at sexual maturity [10], [30].

In order to reduce the PNP elephant population growth rate to zero, this type and level of mortality event would be required at a frequency of approximately three to four years. This gives an indication of the resilience of elephant populations to environmental perturbation. The demographic response of populations to episodic mortality is influenced by the life-history characteristics of the species. Elephant life-history is typical of large-bodied ungulates in that these mammals have long generation times, low fecundity and high adult survival [30], [31]. In elephant populations, a unique combination of life-history traits prolong demographic response to environmental disturbance [30] and maximum population growth rate tends to be maintained until the very limit where forage resources can no longer support the population before density dependent feedbacks occur [1], [32], [33]. Therefore, stochastic mortality alone has the potential to limit short-term population size, but is unlikely to affect population growth over the long-term. However, in combination with density-dependent effects, elephant populations may be limited by environmental catastrophes and the stochasticity brought about by temporal variation in resources. Thus when populations are close to carrying capacity, and background mortalities are higher and fecundities lower than observed here, less intense mortality would be required to achieve a stabilizing effect, and longer intervals between periodic catastrophes could still result in fire-induced mortalities influencing demographics substantially. Catastrophic fires are likely to be rare events with expected return in the order of decades [27]. Therefore in isolation these events will not provide population regulation, but in combination with other stochastic environmental events and density-dependent feedbacks, they may play a role in population limitation. Thus removal of fire from the system in some actively managed nature reserves may not only be detrimental to the vegetation [7], [28], but also the dynamics of herbivore populations where fire mortality may be avoided. These fire events should not be considered as negative catastrophes but instead as integral to the savanna system, with the potential to make infrequent but positive contributions to the regulation of abundant herbivore populations.

Burning during the late dry season, under “hot-fire” conditions when fires can be very intense, can result in catastrophic mortality of large mammal species. Arson fires during these times have the potential to impact not only the vegetation of the area, but also raise welfare concerns over any animals affected by the fire due to the significant stress responses and behavioural changes which may occur. The conservation status and abundance of species will influence fire management requirements. The contribution of fire mortality to abundant game species (e.g. blue wildebeest (*Connochaetes taurinus*), impala (*Aepyceros melampus*)) population dynamics may be less problematic than to that of threatened species such as the black rhino (*Diceros bicornis*), which would conversely require extreme awareness of the need to prevent potentially intense catastrophic fires to ensure minimum mortality impacts. If herbivore populations are fairly stable, even a rare catastrophic fire could cause a shift in population dynamics that, in combination with the current factors causing regulation, may cause a population decline. Therefore, the integration of the conservation implications of intense, hot-fire suppression (i.e. removal of a potential contributing factor to natural population regulation), the welfare concerns of arson fires and burning during “hot-fire” conditions into fire management strategies for conservation areas is important.

## Materials and Methods

### Study site

**Figure 5:**
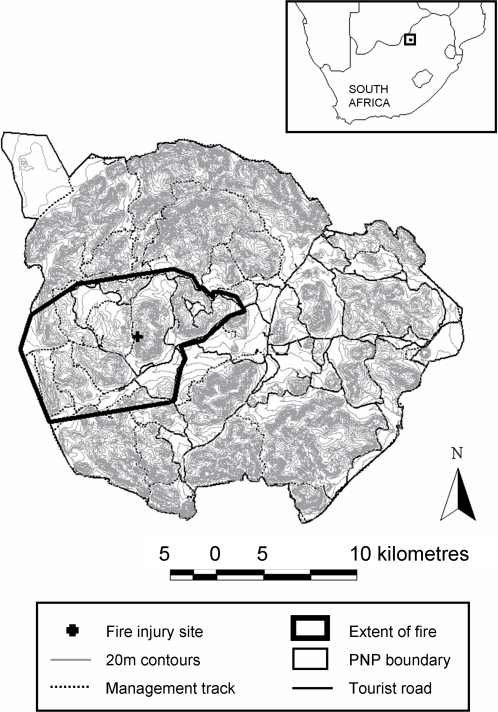
Map of Pilanesberg National Park incorporating 20 m contours, tourist roads, management tracks, the site where elephants were injured in a fire on 21 September 2005 and the approximate extent of the fire.

Pilanesberg National Park (PNP; 25°24′S, 27°08′E; 570 km^2^), North West Province, South Africa, is located within the transition zone of Kalahari Thornveld in the west and Bushveld in the east [34]. The habitat consists mainly of savanna ranging from broadleaf/*Acacia* thickets to open grassland. There are several dams within the Park, one major perennial river system and many ephemeral tributaries and streams. The region has summer rainfall of approximately 630 mm p.a. Geologically, PNP is an extinct volcanic crater formed over 1 200 million years ago and is an example of an alkaline ring complex [35]. The weathering of this complex has created a rugged, hilly landscape, with steep slopes and deep valleys (Figure 5). PNP is open to tourists, but has large “wilderness” areas where there is no tourist access, limited management tracks, and is rarely traversed by people (Figure 5). This wilderness zone comprises approximately half of the total area of PNP. Elephant were introduced to PNP between 1981 and 1998 [36]. As of early September 2005, the PNP population totalled 165 individually identified elephants, of which 37 were independent adult bulls and 128 were part of 18 relatively stable matriarchal family groups. All individuals in the population were known from unique ear notches and tusk configuration. Pre-fire, the population was made up of 86 juveniles under 10 years of age (56 males, 30 females), 23 10–20 year old adults (10 males, 13 females), as well as 29 adult females and 27 adult males between the ages of 20 and 42 (oldest elephants in population). There had been no mortality from old age, with the first expected to occur in around 15 years time [37].

### Behavioural response

We investigated both short-term and long-term responses of the elephants to a fire event that caused elephant injury on the afternoon of 21 September 2005. Short-term responses were investigated over a four and ten-day period pre- and post-fire. Longer-term responses were assessed over three months pre- and post-fire. Prior to the fire, GPS-collars had been fitted to six elephant cows, belonging to different breeding herds within the PNP population. The movement of these elephants (CE03, CE13, CE32, CE61, CE81 and CE88) was assumed to depict the movement behaviour of the breeding herd to which they belonged [14]. Location points were taken at similar times of the afternoon for each cow every day. Members of two of these breeding herds were injured in the fire (CE03 and CE88). CE03 was severely injured, with more than 45% total body surface area (TBSA) burned and CE88 was less severely injured, sustaining burn injuries to approximately 20% TBSA [13]. Analyses pre-fire included data before fire injury occurred, while post-fire analyses included data post-injury.

All statistical analyses in this paper were performed in SPSS 15.0 (SPSS Inc., Chicago, Illinois, USA) with α = 0.05. In the case of parametric tests, assumptions were tested and satisfied. The work was approved by the Animal Ethics Committee of the University of KwaZulu-Natal.

#### Daily displacement

The distance moved by the collared elephants each day (24 hour fixes) was calculated using polylines in the Animal Movement Extension [38] to ArcView 3.2 (ESRI Inc., Redlands, California, USA). We considered this shortest line between the two readings as an index of daily displacement, and refer to this value as daily displacement hereafter.

To test whether there was an initial flight directly after the fire, mean daily displacement of all cows over four days before and after the fire was compared using a paired samples t-test. Four day mean daily displacement of injured cows (CE03, CE88) as well as uninjured cow in close proximity to fire during injury (CE32) was tested with a paired-samples t-test and the same was done for four day mean daily displacement of uninjured cows (CE13, CE61, CE81).

The mean daily displacement during a ten day period before the fire was compared among cows using one-way ANOVA, and the same was done for mean daily displacement for a ten day period, post-flight, after the fire (i.e. day 5–14). A paired-samples t-test was performed on each cow's daily displacement ten days before and post-flight, after the fire. Variability in displacement was assessed using coefficient of variation (CV) for each cow's daily displacement over ten days, pre- and after flight, post-fire; these were contrasted using a paired-samples t-test. We performed the same contrasts of daily displacement from a three month period directly before the fire (21 June 2005–21 September 2005) and after the fire (22 September 2005–22 December 2005).

#### Home range

Each cow's 24-hourly locations for a period of three months pre- and post-fire were mapped in ArcView 3.2. We calculated Kernel home ranges (core home range enclosed by the 50% probability contour and 95% home range enclosed by the 95% probability contour) in animal movement extension SA 2.1 [38], using least-squares cross-validation (LSCV) smoothing. Both 50% core and 95% home ranges were compared before versus after the fire using paired-samples t-tests. Percentage of overlap between the 95% home range before the fire and after the fire was calculated for each cow according to the following equation [39]:
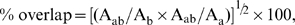
where A_ab_ is the area of overlap between home range before the fire (A_b_) and home range after the fire (A_a_). Percentage overlap data, together with the percentage of locations of each cow in either the wilderness or tourist zones of PNP for three months pre- and post-fire, were used to establish whether a shift in home range had occurred subsequent to the fire and to ascertain if the elephants avoided the tourist zone after the disturbance. A paired-samples t-test was used to compare the percentage location of collared cows in the wilderness zone pre- and post-fire.

An increase in home range size has been reported for elephants in semi-arid environments during the wet season, due to increased access to areas with ephemeral water sources in the wet season [40], [41]. The first spring rains fell in PNP on 4 November, 2005. In order to examine whether there was a change in home range size and location after the rain (and therefore establish whether any change could be attributed to a seasonal shift in home range alone), kernel home range was calculated for 44 day periods before the fire (9 August–21 September 2005), after the fire but before the rain (22 September–4 November 2005), as well as after the rain (5 November–18 December 2005). Areas of 50% and 95% home ranges for each collared cow over these time periods were compared (paired-samples t-test).

Approximately 70% of PNP was burnt during the 2005 dry season. Thus during the late dry season (post-fire and before the rains), the availability of forage was similar throughout the Park in terms of fire-impacted vegetation. We therefore did not consider the post-burn condition of the vegetation as a bias to elephant movement decisions over the study period.

#### Herd fission/fusion

To determine whether the breeding herds showed a ‘fission’ or ‘fusion’ response (i.e. whether breeding herds came together or dispersed, respectively) following the fire, we compared the number of matriarchs (where one matriarch indicates the presence of one herd) seen together in the three-month period pre- and post-fire. A herd's grouping tendency was represented by the percentage fusion, calculated as the number of sightings of a particular herd with other breeding herds, as a percentage of the total number of sightings of that herd. For each herd, percentage fusion was calculated before the fire and after the fire. Because injured and uninjured herds showed opposite fusion trends, a t-test on the difference between pre- and post-fire percentage fusion was used to compare injured and uninjured collared herds. Injured cow fusion response over three months post-fire was further broken down into the first two months post-fire, and the month following that, to examine any change in association pattern on partial recovery from injury.

### Physiological stress response

Severe, persistent stress can cause glucocorticoid levels to increase and remain elevated [42]. The measurement of glucocorticoid metabolite levels in elephant faeces has proven a useful non-invasive way of investigating stress levels in African elephants [12], [43], [44]. A total of 171 fresh (i.e. <6 hours since deposition) elephant faecal samples were collected randomly from the PNP population in the three-month periods before and after the fire. Some samples were collected from known individuals at the time of deposition. Those that were not, were classified according to the approximate age of the elephant from which they came [45], where age was estimated from dung bolus size. Average dung bolus diameter greater than 16 cm was considered to belong to adult bulls; all adult cows in the PNP population, with the exception of one, were below 30 years of age at the time of sample collection, corresponding to dung bolus diameters of approximately 14 cm. For anonymous samples with a bolus >16 cm in diameter, the sample was considered to originate from an adult bull if it was from a site where a single track indicated the presence of a large, solitary elephant. Samples were assigned to cows if they were collected from a site where tracks indicated breeding herd activity and if bolus diameters were between 10–14 cm. Samples with a dung bolus diameter <10 cm were considered to belong to juveniles.

Faecal glucocorticoid metabolite levels were measured using methods involving the use of a corticosterone I^125^ radioimmunoassay (RIA) kit (MP Biomedicals, Costa Mesa, California, USA) [46], [47]. This assay has been validated and used for elephants [12], [47].

Differences in glucocorticoid levels among adult males, adult females and juveniles, pre- and post-fire, were compared using two-way ANOVA. Repeated measures ANOVA was not used because samples before and after the fire were not necessarily, and likely improbably, from the same individuals.

The effect of season on stress levels was examined to establish whether any change in stress hormone level after the fire was consistent with the onset of the first spring rains and thus a seasonal change in stress hormone level. An analysis (two-way ANOVA) of stress hormone levels was carried out over 44 day periods post-fire (i.e. before the rain 22 September–4 November 2005 and after the rain 5 November–18 December 2005). Wet and dry season stress levels for different elephant states (juveniles, adult cows and adult bulls) were compared.

### Demography

The effect of the fire on PNP elephant population size and age structure was assessed by accounting for all mortalities (categorized according to age and sex) and comparing population size and age structure before the fire with that after the fire. A G-test was used to assess if age structure was different, by separating total count data into 10-year and 4-year age classes and comparing the number of elephants in each age class pre- and post-fire.

The potential for fire to limit the population was considered using a probabilistic age and state model [48]. The model was used to calculate population size over 30 years, a time-period relevant for conservation management decision-making, using population data (1) before the fire, and (2) after the fire. The model was also used to determine how often a fire of this nature would need to occur for long-term population growth rate to be reduced to zero over a period of 300 years, to allow for ample reproductive generations and growth. Population growth rate was calculated using projected population size from demographic data before the fire and after the fire, according to the following standard equation for exponential population growth:
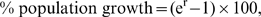
where r = (ln *Nt*_2_–ln *Nt*_1_)/*t* and *Nt*_1_ and *Nt*_2_ are population size at the beginning and end of the time interval in question, respectively; and *t* is the length of the time span in years.

The model incorporated aspects of the life history of individuals and the following important demographic parameters according to acceptable values from the literature: maximum expected lifespan of 60 years [18], [37], female age at sexual maturity of ten years [10], [31], average calving interval for the population of four years [31], [49], age at menopause of 50 years [37], [50] and a 1:1 sex ratio of newborns [31], [49]. These parameters were a slightly conservative estimate of those estimated from past elephant demographic patterns in PNP [10].

The model used was a probabilistic matrix model, where numbers of individuals of different ages were transitioned through specific biological states, i.e. males; sexually immature females; sexually mature, non-pregnant females; pregnant females (in first or second year of pregnancy); females in the first, second, third or fourth year post-parturition. Males were aged but not transitioned through specific states. All parameters other than average calving interval were input as probabilities for each age and state, determined by comparison of input probability value with one obtained from a random number generator that produced a normal distribution of values between 0 and 1. The statistical variation introduced by the probabilistic approach was determined by repeating each simulation 500 times and the means and standard deviations were calculated from these replicate simulations. The population was recorded at the end of each year of a simulation.
